# Synthesis of imidazo-1,4-oxazinone derivatives and investigation of reaction mechanism

**DOI:** 10.3906/kim-2106-28

**Published:** 2021-10-19

**Authors:** Volkan TAŞDEMİR

**Affiliations:** 1 Muradiye Vocational School, Van Yüzüncü Yıl University, Van Turkey

**Keywords:** Cyclization, imidazole, imidazoxazinone

## Abstract

In this study, nine different C-2 aroyl imidazole derivatives were synthesized in a one pot reaction with two steps, and the reduction reactions of these derivatives with NaBH_4_ were carried out under mild conditions. Substitution reaction of obtained imidazo methanol derivatives with chloroacetylchloride reagent and ring reaction of substitution products were investigated. It was determined that 1,4-imidazoxazinone derivative was obtained as a result of the cyclization reaction. The intermediate products obtained during the cyclization reaction were isolated, and the path of the reaction under different conditions was discussed.

## 1. Introduction

Imidazole ring is an essential heterocyclic compound containing two nitrogens in its structure. In literature studies, it is seen that imidazole compounds are used in various fields such as biomimetic catalysts [1–3], medical drugs [4], artificial receptors [5], agricultural chemicals [6], sensors [7], supramolecular ligands [8], and batteries [9]. The imidazole ring contains an acidic proton as well as a nitrogen atom with a basic character. Due to this feature, the zwitterionic structure provides sensitivity like an ionic structure [10]. Thanks to this feature of imidazole compounds, it is possible to break the NH proton in the imidazole ring with various bases and to obtain heterocyclic structures containing imidazole ring by substitution with various reagents [11–14]. Imidazole, a member of the electron-rich azole family, can bind to proteins with weak interactions. Due to such properties, it shows a wide range of biological activity in biological systems through coordination, ion-dipole, cation-π, π-π interaction, and Van der Waals interactions [15,16].

**Figure 1:**
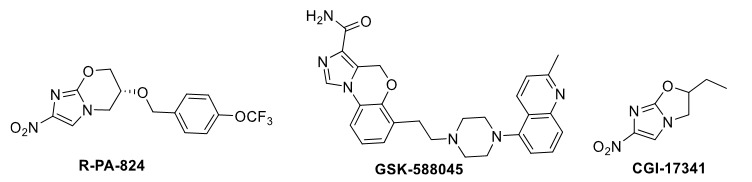
Some important heterocyclic compounds, including imidazoxazine and imidazoxazole

Nowadays, drugs such as oxiconazole, clotrimazole, etc. are drugs that contain imidazole in their structure and are used to treat various diseases [17]. Although imidazole-based heterobicyclic compounds are essential structures, imidazole-2-aroyl derivatives and the structures of these derivatives such as imidazoxazinone derivatives with various reagents have hardly been investigated. Recently, great effort has been put into developing new, effective, biologically active substances to obtain important heterobicyclic molecules such as imidazoxazines. Imidazoxazines (Figure 1) have some critical activities such as tuberculosis (R-PA-824) [18], anxiety, depression, and anticancer (GSK-588045) [19]. Another important imidazole derivative known to have biological activity is imidazoxazole (CGI-17341) [20]. 

**Figure 2:**
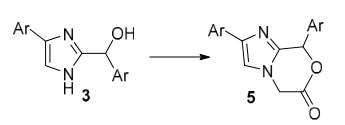
Imidazoxazinone 5.

This study aims to create practical approaches to synthesize heterobicyclic molecules with imidazole rings having different unknown structures and investigate their chemistry in detail. Although there are many examples of imidazole derivatives, the synthesis of imidazoxazinone 5 derivatives (Figure 2), which is not included in the literature, has been investigated. While there are many reaction pathways to obtain imidazole ring, we have utilized the aryglyoxal starting from aryl-acetyl compounds, which was reacted with SeO_2_. Arylglyoxals are very critical starting materials for different types of heterocyclic molecules such as oxiran, β-lactam, pyrrolidine, pyrrole, and pyrazole [21–23]. In addition, we performed the isolation of the intermediate products during the cyclization reactions, and the path followed by the reaction was also discussed.

## 2. Experimental

### 2.1. Materials and methods

Solvents were dried by refluxing with appropriate drying agents and were distilled before use. Melting points were determined using an Electrothermal Gallenkamp apparatus and are uncorrected. FT-IR spectra were obtained in ATR mode using a Thermo Nicolet iS10. Elemental analysis was carried out using a Thermo Scientific Flash 2000. ^1^H-NMR and ^13^C-NMR spectra were recorded using a 400 MHz Agilent using TMS (tetramethylsilane) as the internal standard. All experiments were followed by TLC (thin layer chromatography) using DC Alufolien Kieselgel 60 F254 (Merck) and a Camag TLC lamp (254/366 nm). Commercially available chemicals were purchased from Merck, Aldrich, ABCR and Alfa Easer Co.

### 2.2. General procedure 1 

Acetophenone (1mmol) 1 derivatives were dissolved in 30 mL of 1,4-dioxane in a 100mL flask. SeO_2_ (2.5 mmol) was added and refluxed. The course of the reaction was followed by TLC (thin layer chromatography). The reaction was seen to be finished after 24 h. The reaction was filtered, and 20mL of dissolved ammonium acetate (5 mmol) was added and stirred at room temperature. After determining the completion of the reaction by TLC method, the reaction was filtered with ice water for half an hour and dried. As a result, C-2 aroyl substituted imidazole derivatives 2 were obtained.

### 2.3. General procedure 2

C-2 aroyl substituted imidazole derivatives 2 (1 mmol) were dissolved in 30 mL of methanol, and NaBH_4_ (3 mmol) was added, and it was understood that the reaction was completed after 24 h by TLC method. It was extracted with ethyl acetate and water (30X50). It was dried with MgSO_4_ and evaporated. The column chromatography purified the crude product with eluent ethyl acetate / n-hexane 1/5. The C-2 aroyl substituted imidazolo methanol 3a-i derivatives were obtained. 

#### 2.3.1. Phenyl(4-phenyl-1H-imidazol-2-yl)methanol(3a) [24]

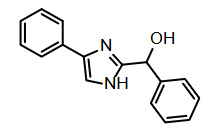



Yield; 73%, Color; white solid. m.p: 170–172°C

FT-IR(ATR cm^–1^): 3210, 3053, 2833, 2678, 2050, 1888, 1608, 1588, 1511, 1496, 1480, 1458, 1435. ^1^H NMR (400 MHz, d-DMSO) δ = 12.10 (s, 1H, N-H), 7.74–7.72 (m, 2H, Ar-H), 7.47–7.45 (m, 3H, Ar-H), 7.34–7.28 (m, 4H, Ar-H), 7.25–7.21 (m, 1H, Ar-H), 7.16–7.12 (m, 1H, Ar-H), 6.27 (d, *J= *2.08 Hz, 1H, OH), 5.79 (s, 1H, CH).^13^C NMR (100 MHz, d-DMSO) δ= 151.3, 143.5, 128.8, 128.5, 127.6, 126.9, 126.4, 124.6, 70.1. LC-MS/MS Anal.Calcd. for C_16_H_15_N_2_O [M+H]: 251.11789, Found: 251.11841.

#### 2.3.2. (4-Methoxyphenyl)(4-(4-methoxyphenyl)-1H-imidazol-2-yl)methanol(3b) [24,25]

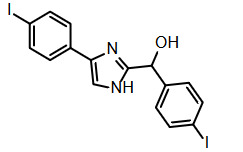



Yield; 80%, Color; white solid. m.p: 166–168°C

FT-IR(ATR cm^–1^): 3162, 3008, 2961, 2835, 2626, 2037, 1609, 1585, 151568, 1511, 1488, 1455, 1425. ^1^H NMR (400 MHz, CDCl_3_) δ = 11.9 (s, 1H, NH), 7.73–7.61 (m, AA’ part of AA’BB’ system, 2H, Ar-H), 7.34-7.32 (m, 3H, Ar-H), 6.87–6.85 (m, BB’ part of AA’BB’ system, 4H, Ar-H), 6.08 (d, *J=* 3.8 Hz, 1H, OH), 5.68 (d, *J= *3.8 Hz, 1H, CH), 3.72 (s, 3H, OCH_3_), 3.70 (s, 3H, OCH_3_). ^13^C NMR (100 MHz, CDCl_3_) δ= 158.8, 158.0, 151.2, 139.7, 135.7, 128.1, 125.8, 114.2, 113.8, 111.5, 69.7, 55.5, 55.4. LC-MS/MS Anal.Calcd. for C_18_H_19_N_2_O_3_ [M+H]: 311.13902, Found: 311.13937.

#### 2.3.3. (4-Iodophenyl)(4-(4-iodophenyl)-1H-imidazol-2-yl)methanol(3c)

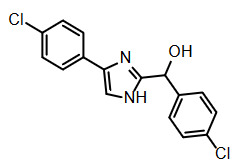



Yield; 40%, Color; red solid. m.p: 270°C

FT-IR(ATR cm^-1^): 3201, 2050, 1980, 1908, 1653, 1587, 1526, 1482. ^1^H-NMR (400 MHz, CDCl_3_) δ = 7.46–7.42 (m, AA’ part of AA’BB’ system, 4H, Ar-H), 7.26–7.23 (m, BB’ part of AA’BB’ system, 2H, Ar-H), 7.02–7.00 (m, 3H, Ar-H), 5.70 (s, 1H, CH). ^13^C NMR (100 MHz, CDCl^3^) δ=150.6, 141.7, 137.4, 137.1, 128.6, 126.5, 93.1, 91.2, 69.4. LC-MS/MS Anal.Calcd. for C_16_H_13_I_2_N_2_O [M+H]: 502.91118, Found: 502.91223.

#### 2.3.4. (4-Chlorophenyl)(4-(4-chlorophenyl)-1H-imidazol-2-yl)methanol(3d) [24]

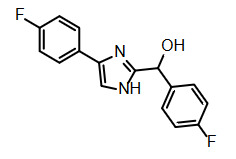



Yield; 75%, Color; red solid. m.p: 160-170°C

FT-IR(ATR cm^–1^): 3148, 2835, 2627, 1609, 1510, 1486, 1448, 1409. ^1^H-NMR (400 MHz, CDCl_3_) δ = 12.18 (s, 1H, NH), 7.74–7.72 (m, AA’ part of AA’BB’ system, 2H, Ar-H), 7.53 (s, 1H, CH), 7.47–7.45 (m, AA’ part of AA’BB’ system, 2H, Ar-H), 7.39–7.33 (m, BB’ part of AA’BB’ system, 4H, Ar-H), 6.37 (s, 1H, OH), 5.77 (s, 1H, CH). ^13^C NMR (100 MHz, CDCl_3_) δ=151.1, 142.3, 132.2, 130.6, 128.8, 128.7, 128.5, 126.3, 69.2. LC-MS/MS Anal.Calcd. for C_16_H_13_CI_2_N_2_O [M+H]: 319.03994, Found: 319.04037.

#### 2.3.5. (4-Fluorophenyl)(4-(4-fluorophenyl)-1H-imidazol-2-yl)methanol(3e)

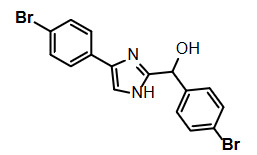



Yield; 73%, Color; pink solid. m.p:155-157°C

FT-IR(ATR cm^–1^): 3148, 3032, 2835, 2628, 2050, 1980, 1609, 1583, 1565, 1509, 1487, 1445. ^1^H-NMR (400 MHz, CDCl_3_) δ = 12.14 (s, 1H, NH), 7.77–7.74 (m, AA’ part of AA’BB’ system, 2H, Ar-H), 7.51–7.48 (m, AA’ part of AA’BB’ system, 2H, Ar-H), 7.46 (s, 1H, CH), 7.17–7.11 (m, BB’ part of AA’BB’ system, 4H, Ar-H), 6.36 (s, 1H, OH), 5.81 (s, 1H, CH). ^13^C NMR (100 MHz, CDCl_3_) δ=163.0, 162.4, 160.6, 160.0, 151.2, 139.7, 139.6, 128.8, 126.5, 126.4, 115.7, 115.5, 115.3, 115.0, 69.3. LC-MS/MS Anal.Calcd. for C_16_H_13_F_2_N_2_O [M+H]: 287.09905, Found: 287.09885.

#### 2.3.6. (4-Bromophenyl)(4-(4-bromophenyl)-1H-imidazol-2-yl)methanol(3f)

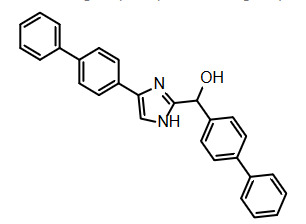



Yield; 65%, Color; red solid. m.p: 155–159°C

FT-IR(ATR cm^–1^): 3139, 2809, 2658, 1716, 1590, 1553, 1514, 1477, 1448, 1406. ^1^H-NMR (400 MHz, CDCl_3_) δ = 12.17 (s, 1H, NH), 7.68–7.66 (m, AA’ part of AA’BB’ system, 2H, Ar-H), 7.53–7.46 (m, 5H, Ar-H), 7.40–7.38 (m, BB’ part of AA’BB’ system, 2H, Ar-H), 6.36 (d, *J=* 3.3 Hz, 1H, OH), 5.75 (d, *J=* 3.3 Hz, 1H, CH). ^13^C NMR (100 MHz, CDCl_3_) δ= 151.0, 142.7, 131.7, 131.4, 129.1, 126.6, 120.7, 119.0, 69.3. LC-MS/MS Anal.Calcd. for C_16_H_13_Br_2_N_2_O [M+H]: 406.93891, Found: 406.93964.

#### 2.3.7. [1,1’-Biphenyl]-4-yl(4-([1,1’-biphenyl]-4-yl)-1H-imidazol-2-yl)methanol(3g)

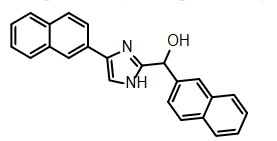



Yield; 57%, Color; brown solid. m.p: 255–259°C

FT-IR(ATR cm^–1^): 3266, 3148, 3055, 3032, 2836, 2621, 2049, 1614, 1583, 1568, 1556, 1510, 1485, 1458, 1444, 1405. ^1^H NMR (400 MHz, d-DMSO) δ = 12.19 (s, 1H, N-H), 7.84–7.82 (m, 2H, Ar-H), 7.67–7.61 (m, 8H, Ar-H), 7.57–7.54 (m, 2H, Ar-H), 7.45–7.41 (m, 5H, Ar-H), 7.35–7.32 (m, 2H, Ar-H), 6.33 (d, *J= *3.2 Hz,1H, OH), 5.85 (d, *J=* 3.2 Hz, 1H, CH). ^13^C NMR (100 MHz, d-DMSO) δ= 151.4, 142.6, 140.5, 140.4, 139.5, 137.9, 131.9, 129.6, 129.4, 127.8, 127.6, 127.5, 127.1, 126.9, 126.7, 125.9, 125.2, 69.9. LC-MS/MS Anal.Calcd. for C_28_H_23_N_2_O [M+H]: 403.18049, Found: 403.18079.

#### 2.3.8. Naphthalen-2-yl(4-(naphthalen-2-yl)-1H-imidazol-2-yl)methanol(3h)

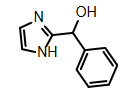



Yield; 40%, Color; yellow solid. m.p: 176–180°C

FT-IR(ATR cm^–1^):3263, 3053, 1718, 1630, 1599, 1507, 1367. ^1^H NMR (400 MHz, d-DMSO) δ = 12.21 (s, 1H, N-H), 8.23 (s, 1H, Ar-H), 7.92–7.81 (m, 8H, Ar-H), 7.62–7.60 (m, 2H, Ar-H), 7.51–7.44 (m, 4H, Ar-H), 6.39 (s, 1H, OH), 5.98 (s, 1H, CH).^13^C NMR (100 MHz, d-DMSO) δ= 151.5, 140.9, 128.8, 133.9, 133.2, 132.8, 132.2, 128.3, 128.1, 127.9, 126.6, 126.3, 125.5, 125.1, 124.1, 121.9, 70.3.LC-MS/MS Anal.Calcd. for C_24_H_19_N_2_O [M+H]: 351.14919, Found: 351.14966.

#### 2.3.9. (1H-Imidazol-2-yl)(phenyl)methanol(3i) [26]

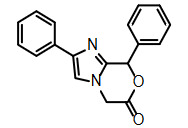



Yield; 58%, Color; white solid. m.p: 196–199°C

FT-IR(ATR cm^–1^): 3163, 3063, 3029, 2891, 2607, 1610, 1584, 1510, 1488,1451. ^1^H NMR (400 MHz, d-DMSO) δ = 11.86 (s, 1H, N-H), 7.37–7.19 (m, 5H, Ar-H), 6.85 (s, 2H, Ar-H), 6.14 (s, 1H, OH), 5.70 (s, 1H, CH). ^13^C NMR (100 MHz, d-DMSO) δ= 150.7, 143.7, 128.4, 127.4, 126.8, 70.0. LC/MS-MS Anal.Calcd. for C_10_H_10_N_2_O [M+H]: 175.08659, Found 175.08705.

### 2.4. General procedure 3

C-2 aroyl substituted imidazolo methanol 3a-h derivatives (1 mmol) were dissolved in dry DCM. Chloroacetylchloride (1 mmol) was added and heated at 45 °C by stirring. It was understood that the reaction was finished after 2 h by TLC method. The reaction flask was filtered and evaporated. Crude product 5a was obtained.

#### 2.4.1. 2,8-Diphenyl-8H-imidazo[2,1-c][1,4]oxazin-6(5H)-one (5a)

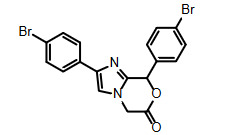



Yield; 87%, Color; light yellow viscose.

FT-IR(ATR cm^–1^): 3148, 2980, 2835, 2639, 1747, 1633, 1601, 1583, 1509, 1486, 1455, 1408, 1342. ^1^H NMR (400 MHz, CDCl_3_) δ = 7.83–7.80 (m, 2H, Ar-H), 7.73–7.70 (m, 2H, Ar-H), 7.43 (s, 1H, Ar-H), 7.35 (s, 1H, CH), 7.29–7.25 (m, 6H, Ar-H), 4.64 (AB sistem, *J =* 15.8 Hz, 1H, CH_2a_), 4.26 (AB sistem, *J=* 15.8 Hz, 1H, CH_2b_). ^13^C NMR (100 MHz, CDCl_3_) δ = 166.7, 144.9, 134.1, 133.5, 130.0, 129.7, 129.3, 129.1, 127.7, 125.9, 125.8, 114.1, 70.2, 41.1. LC/MS-MS Anal.Calcd. for C_18_H_14_N_2_O_2_ [M+H]: 291.11280, Found 291.11392.

#### 2.4.2. 2,8-bis(4-bromophenyl)-8H-imidazo[2,1-c][1,4]oxazin-6(5H)-one(5f)

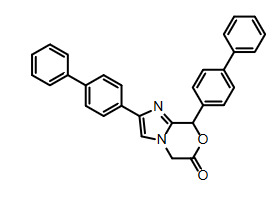



Yield; 60%, Color; light yellow viscose

FT-IR(ATR cm^–1^): 3147, 2833, 2635, 1770, 1633, 1593, 1539, 1485, 1440, 1398, 1310. ^1^H NMR (400 MHz, CDCl_3_) δ = 7.68 (br, 2H, Ar-H), 7.59 (br, 2H, Ar-H), 7.44–7.40 (m, 6H, Ar-H, imidazole CH and CH), 4.62 (AB sistem, J= 13.7 Hz, 1H, CH_2a_), 4.30 (AB sistem, J= 13.7 Hz, 1H, CH_2b_).^13^C NMR (100 MHz, CDCl_3_) δ= 166.7, 144.6, 133.4, 132.6, 132.4, 129.3, 127.4, 124.6, 124.2, 114.6, 69.4, 41.0. GC/MS (e/z) Anal.Calcd. for C_18_H_12_Br_2_N_2_O_2_: 445.93, Found 445.93.

#### 2.4.3. 2,8-di([1,1’-biphenyl]-4-yl)-8H-imidazo[2,1-c][1,4]oxazin-6(5H)-one(5g)

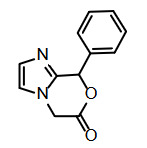



Yield; 55%, Color; green viscose

FT-IR(ATR cm^–1^): 3132, 3030, 2833, 2641, 1746, 1633, 1603, 1485, 1471, 106, 1300. ^1^H NMR (400 MHz, CDCl_3_) δ = 7.92–7.90 (m, 2H, Ar-H), 7.69–7.62 (m, 3H, Ar-H), 7.51–7.45 (m, 3H, Ar-H), 7.36–7.20 (m, 12H, Ar-H, imidazole CH and CH), 4.64 (AB sistem, J= 14.8 Hz, 1H, CH_2a_), 4.26 (AB sistem, J= 14.8 Hz, 1H, CH_2b_).^13^C NMR (100 MHz, CDCl_3_) δ= 166.8, 144.8, 142.8, 142.2, 139.6, 139.4, 133.9, 132.3, 128.8, 128.8, 128.2, 127.9, 127.6, 126.9, 126.2, 124.6, 114.2, 70.0, 41.2. GC/MS (e/z) Anal.Calcd. for C_30_H_22_N_2_O_2_: 442.17, Found 442.17

#### 2.4.4. 8-phenyl-8H-imidazo[2,1-c][1,4]oxazin-6(5H)-one(5i)

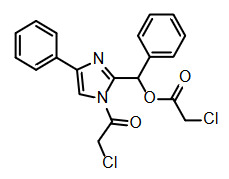



Yield; 68%, Color; light yellow viscose

FT-IR(ATR cm^–1^): 3149, 3059, 2858, 2672, 1766, 1611, 1496, 1456, 1408, 1351. ^1^H NMR (400 MHz, CDCl_3_) δ = 7.65–7.64 (m, 2H, Ar-H), 7.34 (m, 2H, Ar-H), 7.30 (s, 1H, CH), 7.26 (m, 1H, Ar-H), 7.18 (s, 2H, Ar-H), 4.59 (AB sistem, J= 15.7 Hz, 1H, CH_2a_), 4.24 (AB sistem, J= 15.7 Hz, 1H, CH_2b_). ^13^C NMR (100 MHz, CDCl_3_) δ= 166.6, 144.4, 133.3, 130.2, 129.4, 127.6, 118.9, 70.2, 41.0. GC/MS (e/z) Anal.Calcd. for C_12_H_10_N_2_O_2_: 214.07, Found 214.07

#### 2.4.5. (1-(2-Chloroacetyl)-4-phenyl-1H-imidazol-2-yl)(phenyl)methyl 2-chloroacetate (11a)

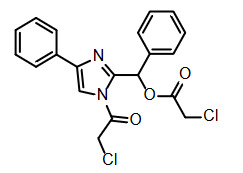



Yield; 88%, Color; grey solid. m.p: 138–140°C

FT-IR(ATR cm^–1^): 3161, 3063, 3034, 2981, 2964, 2940, 1960, 1748, 1606, 1531, 1495, 1450, 1387. ^1^H NMR (400 MHz, CDCl_3_) δ = 8.34 (d, *J=* 0.9 Hz, 1H, Ar-H), 7.81(d, *J=* 8.1 Hz, 2H, Ar-H), 7.48–7.46 (m, 2H, Ar-H), 7.43–7.36 (m, 5H, Ar-H), 7.32–7.30 (m, 1H, CH), 7.28 (s, 1H, Ar-H), 5.14 (AB sistem, *J=* 1.2, 15.8 Hz, 2H, CH_2_), 4.52 (AB sistem, *J=* 1.2, 15.8 Hz, 2H, CH_2_). ^13^C NMR (100 MHz, CDCl_3_) δ= 167.0, 165.5, 147.6, 136.1, 132.6, 129.2, 129.1, 128.9, 128.9, 128.8, 128.3, 125.4, 115.2, 72.8, 44.7, 41.5. LC/MS-MS Anal.Calcd. for C_20_H_16_Cl_2_N_2_O_3_ [M+H]: 403.06107, Found 403.06201.

## 3. Results

**Scheme 1:**
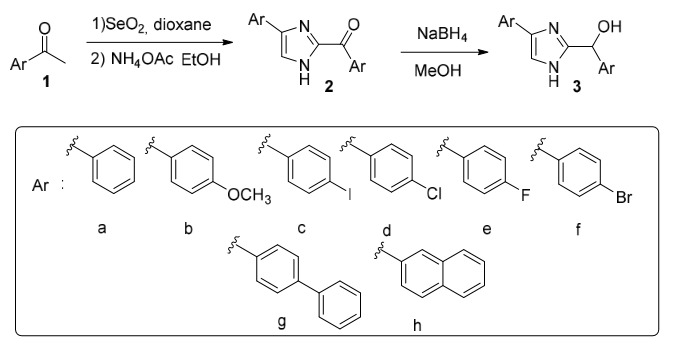
C-2 aroyl substituted imidazolo methanol derivatives.

In this study, for starting compounds, C-2 aroyl substituted imidazolo methanol derivatives **3** shown in Scheme 1 were obtained from C-2 aroyl substituted imidazole derivatives **2,** which is not found in the literature. The synthesis of 1,4-imidazoxazinone derivatives of C-2 aroyl substituted imidazolo methanol derivative compounds with chloroacetylchloride **13** under the various base and solvent conditions was investigated.

**Scheme 2:**
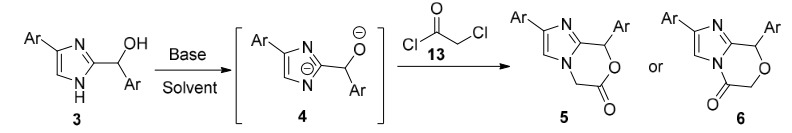
Heterobicyclic imidazoxazinone derivatives.

C-2 aroyl substituted imidazolo methanol **3** derivatives were used as starting compound for obtaining **5** or **6** molecules of heterobicyclic imidazoxazinone derivatives. As shown in Scheme 2, cyclization experiments were carried out on reagents using various bases.

Table 1 shows the experiments performed in the presence of various bases, the reagent used, and the starting compound. To abstract the NH and OH protons in the starting compound **3a** as in the mechanism given in Scheme 2, when sodium hydride (NaH) is used as a base in DMF, compound **4** formed. However, when compound **13** was added later, neither products **5-6** formation nor starting compound **3a** was obtained. No product formation was observed when potassium carbonate (K_2_CO_3_) was used as a base in dichloromethane (DCM). The starting product **3a** was obtained when the starting compound was made directly with acetic acid (AcOH). We know well from previous studies that **4** intermediate products were formed [27]. As a result of the experiments carried out, it is thought that the compound **13** reagent used has two different ends that can react, and the polymeric structures are formed as a result of the reaction of compound **4** formed by the use of inorganic bases.

**Table 1:** Experiments with compound 3a in the presence of various bases.

Compound		Chloroacetylchloride 13	Solvent	Base	Temperature	Result	Time (h)
1	3a	2.8 mmol	DMF	NaH	0°C	ndb	24
2	3a	1 mmol	DCM	K2CO3	Room Temp.	ndb	24
3	3a	1 mmol	AcOH	-	Room Temp.	nra	24
4	3a	1.4 mmol	THF	K2CO3	Room Temp.	ndb	24

nra: No reaction, starting material was recovered. ndb: Non-isolated mixture.

The experiments were concentrated upon obtaining compound **9** from the reaction of compound **13** with the formation of **8** by breaking off the NH proton present in the phenyl (4-phenyl-1H-imidazol-2-il) methanone **7a** with various bases shown in Table 2.

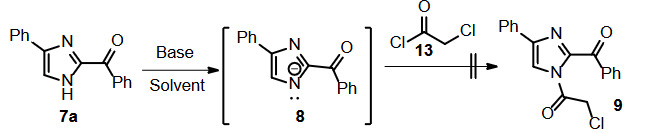



**Table 2:** Trials to obtain molecule 9.

Compound		Chloroacetylchloride	Base	Solvent	Temp.	Result	Time (h)
1	7a	1.4 mmol	NaH (1.6 mmol)	DMF	0 °C	nra	24
2	7a	1.4 mmol	NaH (1.6 mmol)	THF	0 °C	nra	24
3	7a	1.4 mmol	K2CO3 (2.5 mmol)	THF	Reflux	nra	24
4	7a	1.4 mmol	K2CO3 (2.5 mmol)	Acetone	Reflux	nra	24
5	7a	1.4 mmol	TEA (3 mmol)	THF	Room Temp.	nra	24
6	7a	1.4 mmol	-	AcOH	Reflux	nra	24
7	7a	1.2 mmol	TEA (3 mmol)	Pyridine	Reflux	nra	24

nra: No reaction, starting material was recovered.

Table 2 shows the experiments with various solvents and bases to remove the **7a** NH protons of the imidazole compound. Only the starting compound was recovered from the reaction of the molecule **7a** with bases such as NaH, K_2_CO_3_, and TEA, with compound **13**. In the literature, the NH proton can be easily separated [28–31] with bases such as NaH, K_2_CO_3_ and reacted with the appropriate reagent. In the literature, there are some studies in which removal of one of the NH_2_ protons was done using bases such as NaH, K_2_CO_3_ and substitutions with chloroacetylchloride [32–34]. 

**Scheme 3:**
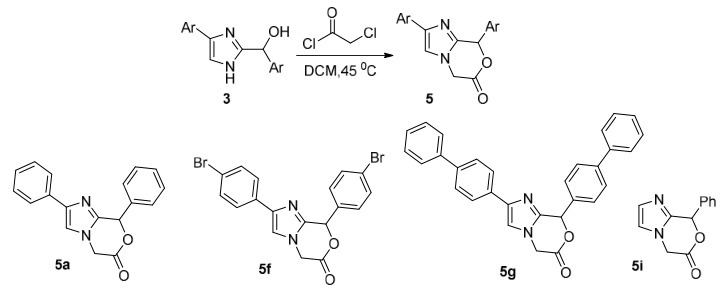
Preparation of imidazoxazinone derivatives.

In the studies performed to obtain imidazoxazinone derivatives in Table 3, no product could be isolated in all other trials except 5a, 8, and 9. It might be due to the presence of more than one reacting group on the imidazole ring, and the presence of two reagent ends in compound **13**. At the same time, it may have caused the formation of different polymeric structures. After this observation, crude NMR was obtained without extraction in all our other trials except 8 and 9 in Table 3. In NMR analysis, the formation of **5a**, the molecule we aimed in reactions 1, 2, 5, 6, and 7, was determined. Due to the isolation problems arising from the lactone structure of the molecule **5a**, the peaks of compound **13**, which is overused with the **5a** molecule, were also observed. In the reaction where compound **13** was used at 1/1 stereometric coefficients (Scheme 3), it was observed that molecule **5a** was synthesized purely without using a base, and there was no chemical shift of molecule **13**. Using the base molecule **13** with a 1/1 stichiometric coefficient, a mixture of molecules was observed, which was not understood in crude NMR. In the reaction conditions obtained by taking crude NMR, it was determined that when the reaction medium was heated at 45 °C in dry DCM for 2 h without using base, high purity targeted molecule was synthesized without leaving starting compounds **3a** and **13**.

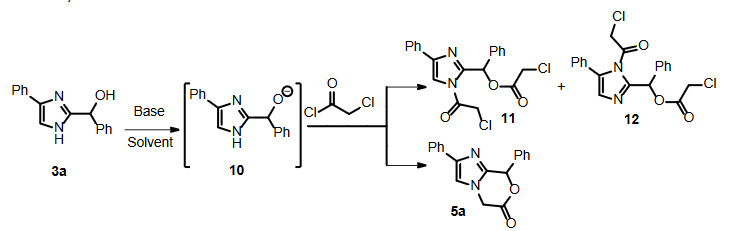



**Table 3:** Synthesis of imidazoxazinone molecule.

Compound		Chloroacetylchloride	Base	Solvent	Temp.	Result	Time
1	3a	1.4 mmol	-	THF	45 °C	5a	15 min
2	3a	1.4 mmol	-	DCM	Room Temp.	5a	2 h
3	3a	1.4 mmol	TEA	DCM	Room Temp.	11 or 12	2 h
4	3a	1 mmol	TEA	DCM	Room Temp.	ndb	1 h
5	3a	1.4 mmol	NaHCO3	MeCN	Room Temp.	5a	30 min
6	3a	1 mmol	-	DCM	45°	5a	2 h
7	3a	1 mmol	-	THF	Room Temp.	5a and 3a (1/3)	2 h
8	3a	1 mmol	-	AcOH	Room Temp.	3a	24 h
9	3a	1 mmol	-	THF	45°C	ndb	2 h

ndb: Non-isolated mixture.

Various cyclization trials were carried out under the reaction conditions shown in Table 3 to obtain imidazoxazinone derivatives, as shown in Scheme 3. As a result, it was observed that the product formed in the reaction of compound **3a** and compound **13** in DCM solvent at a ratio of 1: 1 at 45 °C was imidazoxazinone derivative is compound 5.

In the reaction without the use of a base, it was thought that the OH group would react with the acyl carbonyl [35] and substitute the chloroacetylchloride compound on oxygen, and then the molecule would be cyclized by cleaving the NH proton in the imidazole compound with the appropriate base. As predicted, it was observed that the reaction started when the OH group in the imidazole molecule attacked the electrophilic acyl carbonyl of compound **13,** and the compound **5a** was formed in the solvent medium without using any base as a result of the attack of the second electrophilic group of compound **13** to the methyl chloride with the unshared electron pair of the NH nitrogen in the imidazole through the resonance that occurred in the molecule. It was determined that the **5a** molecule was not degraded by NMR taken at certain intervals within a month. However, introducing a nucleophile such as water, alcohol, etc. causes the molecule to react rapidly.

**Figure 3:**
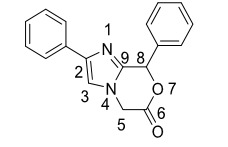
Imidazoxazinone derivative 5a.

When the ^1^H-NMR spectrum is examined over the **5a** compound shown in Figure 3, it is seen that the protons of the C_5_ carbon atom are resonant at 4.26 CH_2b_ and 4.64 CH_2a_ ppm. It is understood from the spectrum that these protons are diastereotopic, interacting with each other, and are part of an AB system. When the interactions of these protons were examined, the interaction value was measured as *J=* 15.8 Hz. It is understood from this value that these protons are in the geminal position, and neighboring groups with these protons have π orbitals [36]. It is in harmony with this information in the molecular structure we propose. The proton of the C_8_ carbon resonated as a singlet at 7.35 ppm due to its electronic environment. When the HSQC spectrum is examined, it has been confirmed that the protons resonating at 4.26 and 4.64 ppm are geminal protons and are bound to the same carbon. It is seen that protons resonating at 4.26 to 4.64 ppm are C_5_ paired with the resonant carbon at 41.1 ppm. On the other hand, it has been confirmed from the HSQC spectrum that the proton resonating in the aromatic field is an aliphatic proton that the C_8_ carbon atom protons do not belong to any aromatic system. It was observed that the proton resonant at 7.43 ppm belongs to the resonant carbon C_8_ at 70.1 ppm. It is seen that the lactone carbonyl carbon of the imidazoxazinone compound is C_6_ resonant at 166 ppm and C_9_, C_2, _and C_3_ carbon atoms are resonant at 144.9, 134.1, and 114.0 ppm, respectively.

## 4. Conclusion

In this work, nine different C-2 aroyl imidazole derivatives were synthesized, and C-2 aroyl substituted imidazolo methanol derivative compounds were obtained as the starting compound in the yield of imidazoxazinone derivatives with suitable reducers. Then, the synthesis of 1,4-imidazoxazinone derivatives with chloroacetylchloride under the various base and solvent conditions was investigated. As a result of the obtained imidazo methanol derivatives and the substitution reaction and ring closure reactions, the synthesis of 1,4 imidazoxazinone derivatives and dichloraacetylchloride derivative molecules was carried out. Structure characterizations for the obtained compound **5** were elucidated by using ^1^H-NMR, ^13^C-NMR, and LC-MS / MS. 

## Supporting information

^1^H-NMR, ^13^C-NMR, HSQC, APT spectrum and LC-MS / MS, GC-MS data are provided in the Supplementary Material section of this article.
